# Acupuncture helps to regain the consciousness of a COVID-19 patient complicated with hypoxic-ischemic encephalopathy: a case report

**DOI:** 10.1007/s10072-020-04980-8

**Published:** 2021-01-07

**Authors:** Bo-Yan Yeh, Yen-Lung Chen, Shih-An Chang, Chung-Shu Lee, Yu-Sheng Chen

**Affiliations:** 1 Department of Chinese Acupuncture and Traumatology, Center for Traditional Chinese Medicine, Chang Gung Memorial Hospital, 5 Fu-Shin St, Kwei-Shan, Tao-Yuan, Taiwan 333; 2 Graduate Institute of Clinical Medical Sciences, Chang Gung University, Taoyuan, Taiwan; 3 Department of Thoracic Medicine, Chang Gung Memorial Hospital, Chang Gung University, School of Medicine, Taipei, Taiwan

Dear Editor,

Complications such as cardiac arrest due to coronavirus disease 2019 (COVID-19) may occur during treatment, and the sequelae of hypoxic-ischemic encephalopathy (HIE) [1] worsens the condition. We report a patient with COVID-19 pneumonia complicated with HIE after cardiac arrest who regained consciousness after early acupuncture therapy.

A 73-year-old male chef with hyperlipidemia and a history of intracerebral hemorrhage was admitted for the deterioration of COVID-19 pneumonia on April 11, 2020. Due to rapidly progressive dyspnea, emergency tracheostomy was performed for the difficult airway, and mechanical ventilation has started since April 13. Empiric antibiotics and medication with potential benefits were administered. On April 27, the patient tested negative for COVID-19 three times with clinical improvement, and ventilator support was removed.

On May 1, the patient developed bradycardia with subsequently pulseless electrical activity (PEA). He was immediately rescued with cardiopulmonary resuscitation, and the return of spontaneous circulation was achieved in approximately 3 min. Arterial blood gas analysis revealed respiratory acidosis, and the mechanical ventilator was reconnected. Antibiotics and inotropic agents were administered for septic shock, but the patient remained unconscious with a Glasgow Coma Scale (GCS) of 8 (E3VTM4). Electroencephalography showed a burst-suppression pattern, and brain magnetic resonance imaging excluded acute infarct and hemorrhage. Hypoxic-ischemic encephalopathy was suggested by the neurologist.

**Fig. 1:**
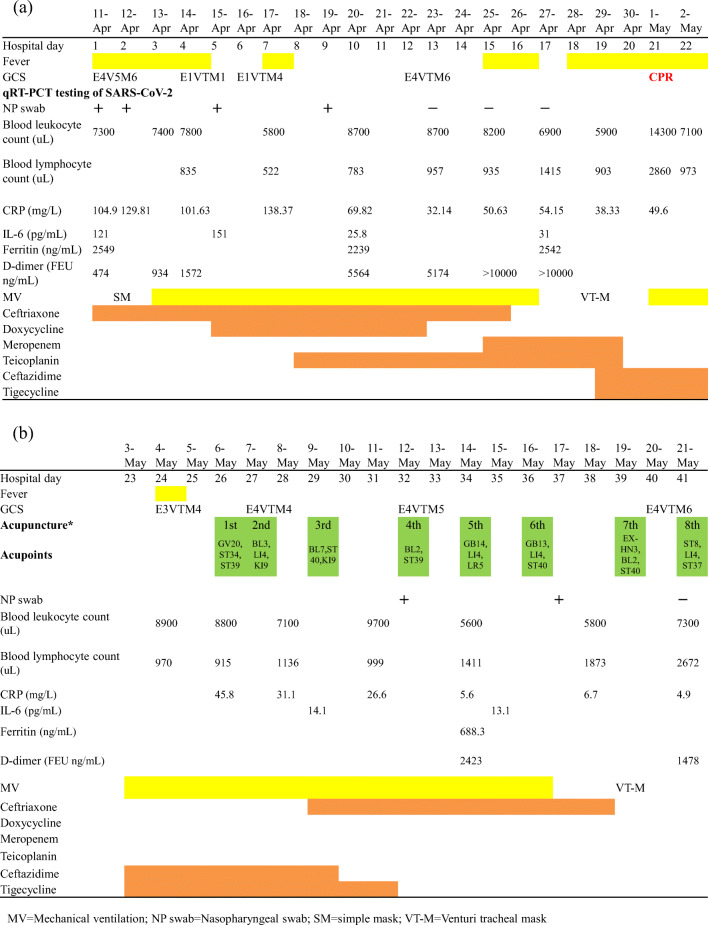
**a** and **b** The clinical course, laboratory finding, and treatment of the case COVID-19

Acupuncture treatment three times a week was initiated on May 6. Acupuncture points were selected based on the principles documented in the literature of classic *Huangdi Neijing* and our clinical experience for patients with HIE. The licensed acupuncturists are physicians of Traditional Chinese Medicine in the medical center who had used acupuncture in their practices for an average of 10 years. Throughout the treatment process, acupuncturists maintained all the concentration on the tip of needles. After the skin was sterilized, acupuncture needles (diameter 0.3 mm; length 40 mm) were inserted without manipulations at GV20 (into the connective tissue layer of scalp), ST34 (into the muscle layer), and ST39 (into the muscle layer) bilaterally the first time. Each needle remained on the interface of the tissue firmly. After the insertion of needles, we ensured the achievement of De Qi by the fine changes in the radial pulse. The needles were retained for 30 min. The detailed regimen of the acupoints and clinical course were shown in Fig. 1.

**Fig. 2:**
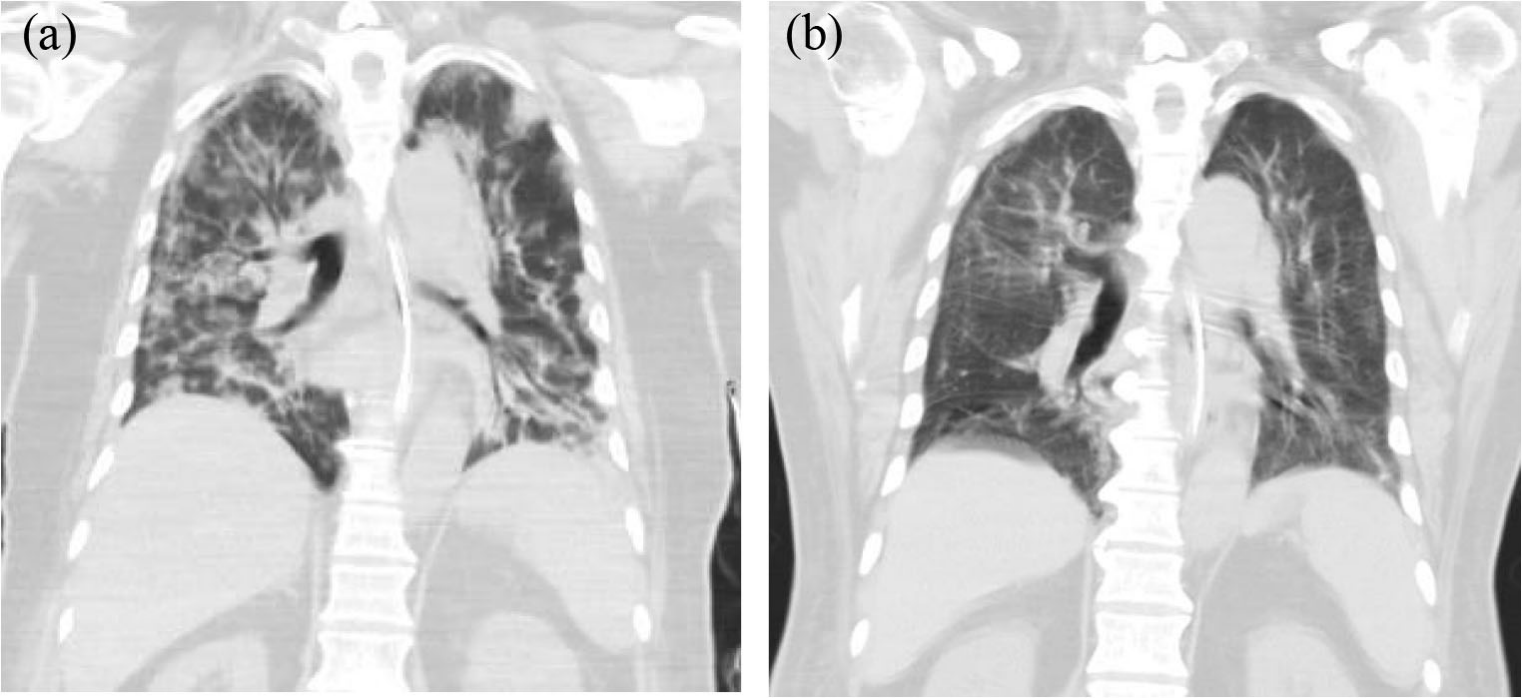
The follow-up chest CT scan of the patient. **a** The CT Scan on April 16 showed patchy ground glass opacity infiltrations distributed diffusely in bilateral lungs. **b** The CT scan on June 2 showed further regression of ground glass opacity and consolidations in the bilateral lungs

After the first acupuncture therapy, intermittent spontaneous eye-opening and limb movements were noticed. The patient gradually regained consciousness, with an increased GCS of 10 (E4VTM5) after 1-week acupuncture therapy. He was weaned off the ventilator at the end of 2-week acupuncture therapy. Antibiotics were also discontinued. Through 3-week management, the GCS score was improved to E4VTM6, and he could perform simple body movements in response to verbal commands. He started to receive bedside rehabilitation and was released from isolation. The follow-up chest computed tomography (CT) of the patient was shown in Fig. 2.

The outcome of patients with severe COVID-19 pneumonia who had an in-hospital cardiac arrest was poor; especially the initial rhythm is asystole or PEA [2]. The electroencephalography report showed a burst suppression pattern suggesting HIE, which pattern indicated the poor prognosis for recovery following HIE [3]. These sequelae implied that the recovery of consciousness would be difficult.

According to our clinical experience, acupuncture has shown benefits for patients who suffer from HIE after rescuing with cardiopulmonary resuscitation. Studies showed that acupuncture treatment for HIE works through many molecular pathways to prevent cell apoptosis and further damage [4]. The evidence supports the potential benefit of acupuncture.

From an acupuncturist’s perspective, acupuncture works through regulating Qi and blood balance and their distribution. The strategy of acupuncture is determined to restore the imbalance of Qi and blood, confirmed by fine changes in the radial pulse. This method has been illustrated that acupuncture could affect the radial pulse patterns [5].

The concept of harmonizing of Qi and blood could be regard as modulating immune function. The research found that anti-inflammatory response occurs in sepsis in attempt to balance the pro-inflammatory response [6], showing that the immunological equilibrium is important to prevent the development of multiple organ failure. Evidence showed that acupuncture could modulate immune function through a complex neuro-endocrino-immunological network of actions with anti-inflammatory effects [7], confirming that acupuncture improve the immune dysfunction in sepsis condition. This patient was at high risk of severe disease on the basis of old age, underlying conditions and laboratory findings. The improvement in consciousness and laboratory findings after acupuncture intervention were observed, suggesting that acupuncture could serve as an alternative method integrating with conventional therapy, providing us a reason for further research.
